# 维奈克拉联合多药化疗治疗初诊未明系列急性白血病的疗效及安全性分析

**DOI:** 10.3760/cma.j.cn121090-20240419-00149

**Published:** 2025-02

**Authors:** 婷 罗, 怡然 方, 文洁 刘, 倩 孙, 佩 徐, 鸣 洪, 思轩 钱

**Affiliations:** 1 南京医科大学第一附属医院、江苏省人民医院血液科，南京 210029 Department of Hematology, First Affiliated Hospital of Nanjing Medical University, Nanjing 210029, China; 2 南京医科大学附属泰州人民医院，泰州 225300 Taizhou People's Hospital, Nanjing Medical University, Taizhou 225300, China

**Keywords:** 维奈克拉, 未明系列急性白血病, 抗肿瘤联合化疗方案, Venetoclax, Acute leukemia of ambiguous lineage, Anti-tumour combination chemotherapy programme

## Abstract

**目的:**

探讨维奈克拉（VEN）联合多药化疗治疗初诊未明系列急性白血病（ALAL）患者的疗效及安全性。

**方法:**

回顾性分析2021年6月至2024年7月在江苏省人民医院住院治疗的13例初诊ALAL患者的临床资料，13例患者的初始诱导治疗方案均为以VEN为基础联合多药化疗，其中VAA+P（VEN+阿扎胞苷/地西他滨+阿柔比星+醋酸泼尼松）方案治疗8例，V+IA（VEN+伊达比星+阿糖胞苷）方案治疗5例，FLT3突变患者联合FLT3抑制剂，Ph^+^患者联合酪氨酸激酶抑制剂治疗。分析患者的总生存（OS）期、无病生存（DFS）期及不良反应。

**结果:**

根据WHO造血与淋巴组织肿瘤分类第五版关于ALAL的免疫表型定义，13例ALAL患者包括T/髓系混合表型急性白血病（MPAL）4例，B/髓系MPAL 7例，ALAL-非特指型2例。其中4例B/髓系MPAL患者Ph（+），属ALAL特定的基因异常组别。3例患者存在FLT3突变（FLT3-TKD突变1例，FLT3-ITD突变2例）。于巩固治疗前评估VEN联合用药方案的诱导治疗效果：13例患者均获完全缓解（CR）。在随后的巩固治疗过程中，1例患者放弃治疗失访，9例患者进行异基因造血干细胞移植（allo-HSCT），其中4例因移植后并发症死亡，5例无病生存。3例患者（年龄≥70岁）按原方案巩固治疗，其中2例无病生存，1例因白血病中枢神经系统浸润死亡。13例患者的中位OS期未达到，75％位OS期为12.0个月，12个月累积OS率为64.5％，所有患者的中位DFS期未达到，75％位DFS期为8.2个月，12个月累积DFS率为67.1％。VEN联合方案诱导治疗期间及治疗后所有患者均发生3级或4级血液学不良反应，包括中性粒细胞减少和血小板减少。所有患者经初始诱导治疗后造血功能恢复且无致死性大出血发生；无患者出现肿瘤溶解综合征及神经系统不良反应；同时无3级及以上脏器不良反应发生（患者治疗前基础病除外）。

**结论:**

VEN联合多药化疗治疗初诊ALAL的疗效值得肯定且治疗的相关不良反应可耐受。

## Abstract (en)

**Objective:**

To evaluate the efficacy and safety of venetoclax in combination with multidrug chemotherapy in patients with newly diagnosed acute leukemia of ambiguous lineage（ALAL）.

**Methods:**

A retrospective analysis of clinical data was performed on patients with newly diagnosed ALAL who were hospitalized at Jiangsu Provincial People's Hospital from June 2021 to July 2024. Of the 13 patients who received initial induction therapy with venetoclax combined with multidrug chemotherapy, 8 received VAA+P regimen, and 5 received V+IA regimen. Patients with FLT3 mutation were treated with FLT3 inhibitor, and Ph^+^ patients received an additional tyrosine kinase inhibitor. Overall survival（OS）, disease-free survival（DFS）, and adverse events were analyzed.

**Results:**

According to the World Health Organization 5th edition of the classification of hematolymphoid tumors, the immunophenotypes were T/myeloid mixed-phenotype acute leukemia（MPAL）（*n*＝4）, B/myeloid MPAL（*n*＝7）, and ALAL- not otherwise specified（*n*＝2）. Of the seven patients with B/myeloid MPAL, four were Ph^+^ and belonged to the group with specific gene abnormalities of ALAL. Three patients had FLT3 mutation（one with FLT3-TKD mutation and two with FLT3-ITD mutation）. Prior to the second course of consolidation therapy, the efficacy of venetoclax induction therapy was evaluated, and a complete response rate of 100％ was achieved in 13 patients. In the subsequent consolidation therapy phase, one patient discontinued treatment and was lost to follow-up; nine patients underwent allogeneic hematopoietic stem cell transplantation, four of whom died due to posttransplant complications and five achieved DFS. Of the three patients（≥70 years old）who received consolidation therapy as before, two achieved DFS and one died due to central nervous system leukemia. The median OS time was not reached in 13 patients; the 75th percentile survival time was 12.0 months, with a 12-month cumulative survival rate of 64.5％. The median DFS time was not reached in all patients; the 75th percentile DFS time was 8.2 months, with a 12-month cumulative DFS rate of 67.1％. All patients experienced grade 3 or 4 hematologic toxicity, including neutropenia and thrombocytopenia, during and after induction therapy. All patients recovered hematopoietic function after the initial induction therapy, with no fatal hemorrhage, tumor lysis syndrome, neurological adverse events, or grade 3 or higher organ toxicity, excluding preexisting conditions.

**Conclusion:**

Venetoclax in combination with multidrug chemotherapy was effective and associated with tolerable adverse reactions in patients with newly diagnosed ALAL.

未明系列急性白血病（acute leukemia of ambiguous lineage，ALAL）是指一部分急性白血病的原始细胞缺乏向单一造血系列分化的特征，发展为一组罕见的、异质性强的恶性血液系统疾病。鉴于临床和免疫表型特征有重叠，第五版《WHO造血与淋巴组织肿瘤分类》（WHO-HAEM5）将ALAL和混合表型急性白血病（MPAL）归为同一个类别。ALAL根据免疫表型定义分为5类：①B/髓系MPAL；②T/髓系MPAL；③罕见类型MPAL；④ALAL-非特指型（NOS）；⑤缺乏系列特异性抗原的急性未分化细胞白血病（AUL）[1]。既往文献报道，急性淋巴细胞白血病（ALL）样治疗方案相较于急性髓系白血病（AML）样治疗方案或“混合”方案（ALL+AML）可改善ALAL特别是MPAL的结局，但一部分患者仍发展为复发难治性ALAL[2]–[6]。

维奈克拉（VEN）是一种B细胞淋巴瘤因子-2（BCL-2）抗凋亡蛋白抑制剂[7]，可特异性结合BCL-2蛋白，阻断其与其他促凋亡蛋白的结合，通过释放促凋亡蛋白恢复细胞内源性凋亡途径，致癌细胞凋亡[8]。VEN已在各种血液系统恶性肿瘤中表现出临床疗效，且现已批准用于AML和慢性淋巴细胞白血病（CLL）的治疗[9]–[10]。除了AML和CLL外，越来越多的研究提出在ALL患者中使用VEN的可行性[11]–[13]。

一些病例报告显示以VEN为基础的联合方案在ALAL中具有较好的治疗效果[14]–[23]，但其疗效及安全性尚未在ALAL患者中得到大规模研究证实。在本研究中，我们回顾性分析了江苏省人民医院既往初诊的MPAL及ALAL-NOS患者基于VEN联合化疗方案的治疗效果，现就其有效性和安全性报道如下。

## 病例与方法

1. 病例资料：以2021年6月至2024年7月在江苏省人民医院住院治疗的13例初诊ALAL患者为研究对象，回顾性分析患者的临床资料。13例患者的诊断符合WHO-HAEM5免疫表型定义，所有患者均根据骨髓细胞形态学、流式细胞术免疫表型分析、细胞遗传学、分子生物学（MICM）进行诊断分型并确诊[1]。

2. 治疗方法：13例患者的初始诱导治疗是以VEN为基础联合多药化疗，所有患者均签署了使用VEN联合方案的知情同意书。诱导治疗包括两种不同的VEN联合用药方案：5例年轻及体能状态良好的患者应用V+IA（VEN+伊达比星+阿糖胞苷）方案，8例老年或初诊体能状态差的患者应用VAA+P（VEN+阿扎胞苷/地西他滨+阿柔比星+醋酸泼尼松）方案。其中，合并FLT3突变患者联合应用FLT3抑制剂（吉瑞替尼或索拉菲尼），合并BCR∷ABL突变患者联合应用酪氨酸激酶抑制剂（达沙替尼或氟马替尼）治疗。为避免肿瘤溶解综合征，VEN采用爬坡治疗，具体用法为100 mg，第1天；200 mg，第2天；400 mg，第3～21天；患者化疗后骨髓抑制期内发生粒细胞缺乏（粒缺）时，依据《血液病/恶性肿瘤患者侵袭性真菌病的诊断标准与治疗原则》[24]，联合应用泊沙康唑预防真菌感染，联合泊沙康唑时VEN减量至100 mg/d。

诱导治疗的联合用药方案中除VEN外其他药物的用法如下：VAA+P方案：阿扎胞苷75 mg·m^−2^·d^−1^，分别于2个部位皮下注射，第1～7天；地西他滨20 mg·m^−2^·d^−1^，静脉滴注，第1～5天；阿柔比星10 mg·m^−2^·d^−1^，静脉滴注，第1～4天；醋酸泼尼松1 mg·kg^−1^·d^−1^，口服，第1～14天，第15天开始可降至1/3剂量用药，后逐渐减量至停用，共用28 d。V+IA方案：伊达比星12 mg·m^−2^·d^−1^，第1～3天；阿糖胞苷100 mg·m^−2^·d^−1^，第1～7天。

参考《成人急性髓系白血病（非急性早幼粒细胞白血病）中国诊疗指南（2023年版）》[25]，所有患者的巩固治疗方案如下：以VAA+P方案诱导治疗的患者，如诱导治疗取得完全缓解（CR），继续原方案巩固治疗；以V+IA方案诱导治疗的患者，取得CR后，巩固治疗方案为VEN+大剂量阿糖胞苷（3 g/m^2^，每日2次，第1～3天）。巩固治疗过程中，合并FLT3突变患者继续联合应用FLT3抑制剂（吉瑞替尼或索拉菲尼），合并BCR∷ABL突变患者继续联合应用酪氨酸激酶抑制剂（达沙替尼或氟马替尼）治疗。13例患者中，仅例7因心电图QTc间期延长，酪氨酸激酶抑制剂由达沙替尼更换为氟马替尼，后因中枢神经系统白血病复发，再次调整为达沙替尼治疗，其余患者均按照上述方案进行。

治疗期间定期监测血常规和肝肾功能，针对药物引起的骨髓抑制，采取输注相应血制品及使用促造血药物：所有患者骨髓抑制期内HGB<60 g/L或出现明显症状时输注悬浮少白红细胞，PLT<20×10^9^/L或出现出血倾向时输注血小板。当患者出现发热或寒战合并感染时，常规进行血培养及病原学检测，按照经验及病原学结果予抗生素/抗真菌药物治疗。

3. 疗效和不良反应评估：所有患者治疗前完成以下检查：血细胞计数及分类，心、肝、肾功能检查，骨髓细胞形态学，流式细胞术免疫表型，染色体核型分析及二代测序基因突变检测。在VEN联合方案诱导治疗1个疗程结束后、巩固治疗前进行疗效评估：疗效判定分为：①CR：骨髓三系造血恢复，原始细胞<5％；外周血无原始细胞；无髓外白血病；外周血ANC>1.0×10^9^/L且PLT>100×10^9^/L；4周内无复发。②CR伴血液学不完全恢复（CRi）：外周血ANC≤1.0×10^9^/L和（或）PLT≤100×10^9^/L，其他满足CR的标准。③未缓解（NR）：未达到CR或CRi。疾病复发指已取得CR的患者外周血或骨髓又出现原始细胞（比例>5％），或出现髓外疾病。采用流式细胞术检测微小残留病（MRD）、PCR检测基因突变及融合基因评估患者各个疗程后的深度缓解情况。难治性疾病定义为治疗结束未能取得CR/CRi。总生存（OS）期为开始应用VEN联合方案诱导治疗至患者死亡或随访截止时间（失访患者OS期为初始治疗到末次随访时间）。无病生存（DFS）期为获得CR的患者，从获得CR之日起至复发或死亡的时间。药物不良反应根据《常见不良反应事件评价标准（CTCAE）》5.0版进行报告和分级。

4. 随访：随访截止日期为2024年7月21日。末次随访患者转归/疾病状态包括疾病持续缓解、死亡、失访。随访方式包括查阅患者住院及门诊病历、电话随访。其中1例患者失访。

5. 统计学处理：采用IBM SPSS Statistics 25软件分析数据。

## 结果

1. 一般临床特征：13例ALAL患者中，男8例，女5例，中位年龄55（27～77）岁。根据WHO-HAEM5免疫表型定义，13例ALAL患者包括MPAL 11例（T/髓系MPAL 4例，B/髓系MPAL 7例），ALAL-NOS 2例。其中例4骨髓免疫分型提示早期前体T急性淋巴细胞白血病（ETP-ALL）合并母细胞性浆细胞样树突状细胞肿瘤（BPDCN）。通过文献复习，我们发现同时显示ETP-ALL和明显的浆细胞样树突状细胞（pDC）增殖的病例极其罕见，且并没有将其归为某一类别。考虑到pDC通常与髓系恶性肿瘤有关，ETP-ALL合并BPDCN的肿瘤干细胞向淋巴、髓系或树突状细胞谱系渐进发展，我们认为，该例患者按照WHO综合诊断可考虑混合细胞白血病，故将其暂时归类为T/髓系MPAL[26]。13例ALAL患者中4例患者Ph（+），属ALAL特定的基因异常组别，均为B/髓系MPAL患者；3例患者存在FLT3突变。化疗前中位WBC为9.33×10^9^/L，中位骨髓原始细胞比例为74.4％。13例患者二代测序共检出42个基因突变，详见表1。所有患者的骨髓免疫表型结果见表2。

**表1:** 13例初诊未明系列急性白血病（ALAL）患者的临床资料

例号	年龄（岁）	性别	诊断	初诊WBC（×10^9^/L）	初诊骨髓原始细胞比例（％）	染色体核型	基因突变（二代测序）
1	77	男	T/髓MPAL	2.58	45.2	46,XY	FLT3-TKD、SRSF2、NRAS、EZH2、RUNX1、TET2
2	30	女	T/髓MPAL	3.71	55.2	46,XX,del（6）（q23）,del（12）（p12）[3]/46,idem,del（11）（p12）[2]/46,XX[7]	ASXL2、PHF6、CCND2、CREBBP、JAK3、NCOR2、NOTCH1
3	27	男	T/髓MPAL	1.80	26.4	46,XY	HOX11L2、WT1、CEBPA
4	55	女	T/髓MPAL	5.56	92.0	46,XX	阴性
5	63	男	B/髓MPAL	10.56	69.0	46,XY,t（9;22）（q34;q11）[7]/46,idem,add（12）（q24）[1]/46,idem,add（5）（q35）[1]/46,idem,t（4;5）（q35;q22）[1]	EZH2、RUNX1、ARID1B、JAK2、SMARCA2、WT1
6	36	男	B/髓MPAL	9.33	95.0	48,XY,+8,t（9;22）（q34;q11）,+21[6]/46,XY[4]	SETD2
7	75	男	B/髓MPAL	19.10	77.6	46,XY,add（8）（p23）,add（9）（p24）,t（9;22）（q34;q11）[3]/46,XY[7]	WT1
8	36	男	B/髓MPAL	25.21	80.0	46,XY,t（9;14）（q34;q32）,ins（22;9）（q11;q34）[9]/46,XY[1]	DNM2、FAT3
9	70	女	B/髓MPAL	93.93	84.8	46,XX	FLT3-ITD、DNMT3A、WT1
10	52	男	B/髓MPAL	19.96	61.2	45,X,-Y,t（8;21）（q22;q22）,de1（9）（q31）[6]/46,XY[1]	FLT3-ITD、CSF3R、EP300、JAK2、SMC1A、WT1
11	70	女	B/髓MPAL	2.45	82.4	47,XX,+8,t（8;21）（q22;q22）[2]/47,idem,add（15）（q26）[7]/46,XX[1]	KIT、STAG2、WT1
12	32	男	ALAL-NOS	0.71	74.4	46,XY	EZH2、FAT4、KMT2D、RAD21、TTN、CIITA、ETV6、KMT2C、KMT2D、PTPN1、DIS3、EPPK1、TTN、WT1
13	57	女	ALAL-NOS	20.08	72.8	46,XX	ASXL1、DNMT3A、IDH2、KRAS、DNMT3A、WT1

**注** MPAL：混合表型急性白血病；NOS：非特指型

**表2:** 13例初诊未明系列急性白血病患者骨髓免疫表型结果

例号	免疫表型
1	群①LAIP：表达TDT、cCD79a、cCD3、CD34、CD117、HLADR、CD13、CD15、CD38、CD2、CD7、CD99；部分表达CD36；不表达cMPO 群②LAIP：表达cMPO、HLA-DR、CD33、CD13、CD11b、CD15、CD38、CD66c、CD64、CD36、CD14、CD11c、CD2
2	LAIP：表达cCD3、CD33、CD38hi、CD81、CD58、CD71、CD7hi、CD5dim、CD99、CD1a；部分表达cMPO、CD34、TDT
3	LAIP：表达cCD3dim、CD7、TDT、CD2dim、CD117、CD34、CD13、HLA-DR、CD33、CD38、CD58、CD81、CD66c、CD56；不表达cMPO、CD99
4	群①LAIP：表达cCD3、TDT、CD34、CD99、CD7hi、CD13、CD5dim、CD81、CD71、CD33、CD38、CD58；不表达CD1a、CD48 群②LAIP：表达HLA-DR、CD13、CD5dim、CD7、CD123hi、CD38、CD303、CD304、CD48、CD4；部分表达CD33、CD81、CD71
5	LAIP：表达CD34、CD117dim、HLA-DR、CD13、CD19、CD81、CD38、CD58、CD22dim；部分表达cCD79a、CD33、CD10、CD9、CD66c
6	群①LAIP：表达cCD79a、CD34、CD13、CD33、HLA-DR、CD10、CD19、CD58、CD81、CD9、CD56、CD66c、CD38；部分表达CD22、CD71 群②LAIP：表达cMPO、cCD79a、CD34、CD13、CD33、HLA-DR、CD10、CD19、CD20dim、CD58、CD81、CD9、CD56、CD66c、CD38；部分表达CD22、CD71
7	群①LAIP：表达cCD79a、CD34、HLA-DR、CD20、CD38、CD19、CD22、CD81、CD71；部分表达CD13、CD33 群②LAIP：表达cMPO、cCD79a、CD34、CD11b、CD13、CD33、HLA-DR、CD38、CD19、CD22、CD20、CD81、CD71；部分表达CD11c
8	群①LAIP：表达cCD79a、CD34、CD117、HLA-DR、CD33、CD13、CD38、CD81、CD123、CD58、CD22、CD19、CD10、CD11c；部分表达CD66c、CD9；不表达CRLF2 群②LAIP：表达cCD79a、CD10、CD19、CD22、CD81、CD38、HLA-DRdim；部分表达CD9；不表达CRLF2
9	LAIP：表达cMPO、CD117、CD34、CD13、CD33、HLA-DR、CD123、CD38、CD19、CD81、CD36、CD71、CD7、cCD79；部分表达CD22、CD11c
10	LAIP：表达cMPO、cCD79a、CD117、CD34、CD19、HLA-DR、CD13、CD33、CD38、CD56、CD71、CD11c、CD58、CD81；部分表达CD123
11	LAIP：表达cMPO、cCD79a、CD117、CD34、CD13、HLA-DR、CD33、CD123、CD19、CD22dim、CD58、CD81、CD38、CD7；部分表达CD11b、CD10、CD11c
12	LAIP：表达CD117、CD34、HLA-DR、CD13dim、CD33、CD19dim、CD38、CD7、CD58、CD71dim；部分表达cCD79a、CD56、CD11c；不表达cMPO
13	LAIP：表达CD117、CD34、CD33、CD38、CD81、CD58、CD11c、CD56、CD7hi；部分表达cCD79a、HLA-DR、CD11b、CD123、CD19；不表达cMPO、cCD3、TdT

**注** LAIP：白血病相关免疫表型

2. 疗效评价：13例患者的初始诱导治疗均以VEN为基础联合多药化疗，其中VAA+P方案治疗8例，例1的联合方案中去甲基化药物为地西他滨，其余7例均为阿扎胞苷；V+IA（VEN+伊达比星+阿糖胞苷）方案治疗5例。FLT3突变患者联合FLT3抑制剂，Ph^+^患者联合酪氨酸激酶抑制剂治疗。巩固治疗前评估VEN联合方案诱导治疗效果：13例患者首疗程诱导治疗后CR率为100％，在随后的巩固治疗过程中，1例患者放弃治疗失访，9例患者进行异基因造血干细胞移植（allo-HSCT），其中4例因移植后并发症死亡，5例无病生存。3例患者（年龄≥70岁）按原方案巩固治疗，其中2例无病生存，1例因白血病中枢神经系统浸润死亡。13例患者的中位OS期未达到，75％位OS期为12.0个月，12个月累积生存率为64.5％，所有患者的中位DFS期未达到，75％位DFS期为8.2个月，12个月累积DFS率为67.1％。除放弃治疗的1例患者外，其余12例患者所有疗程后骨髓形态学均CR。流式细胞术检测12例患者MRD，所有疗程后均持续阴性。所有患者初诊时存在的基因突变及融合基因（包括FLT3-ITD、FLT3-TKD、BCR∷ABL、WT1、H0X11L2、AML1∷ETO、MLL-PTD）转阴情况详见表3。

**表3:** 维奈克拉（VEN）联合多药化疗治疗13例初诊未明系列急性白血病（ALAL）患者的疗效及转归

例号	年龄（岁）	诊断	诱导治疗方案	巩固治疗方案	骨髓形态	MRD^d^	突变及融合基因（1个疗程后）	突变及融合基因（2个疗程后）	移植	转归	OS期（月）
1	77	T/髓MPAL	VAA+P+吉瑞替尼	同方案8个疗程	CR	–	FLT3-TKD 0.16％	–	否	存活	37.2
2	30	T/髓MPAL	V+IA	VEN+Ara-C 1个疗程	CR	–	WT1（–）	–	是	存活	32.4
3	27	T/髓MPAL	V+IA	VEN+Ara-C 1个疗程	CR	–	WT1（–）、H0X11L2（–）	–	是	存活	13.9
4	55	T/髓MPAL	VAA+P	同方案3个疗程	CR	–	WT1（–）	–	是	死亡	10.0
5	63	B/髓MPAL	VAA+P+氟马替尼	同方案3个疗程	CR	–	BCR∷ABL 0.026％	–	是	死亡	12.0
6	36	B/髓MPAL	VAA+P+达沙替尼	同方案1个疗程	CR	–	BCR∷ABL（–）	–	是	死亡	5.3
7	75	B/髓MPAL	VAA+P+达沙替尼	同方案3个疗程 VAA+P+氟马替尼2个疗程VEN+阿扎胞苷+达沙替尼2个疗程	CR	–	BCR∷ABL（–）	–	否	死亡	25.3
8	36	B/髓MPAL	VAA+P+达沙替尼	未知^a^	CR	–	BCR∷ABL（–）	–	是	存活	26.9
9	70	B/髓MPAL	VAA+P+索拉菲尼	未知^b^	CR^c^	/	/	/	否	未知	13.6（至随访）
10	52	B/髓MPAL	V+IA+索拉菲尼	VEN+Ara-C+索拉菲尼 1个疗程	CR	–	FLT3-ITD（–）、WT1（–）、AML1∷ETO（–）	–	是	死亡	7.1
11	70	B/髓MPAL	VAA+P	同方案3个疗程	CR	–	WT1（–）、AML1∷ETO（–）、MLL-PTD（–）	–	否	存活	9.8
12	32	ALAL-NOS	V+IA	VEN+Ara-C 2个疗程	CR	–	WT1（–）	–	是	存活	7.1
13	57	ALAL-NOS	V+IA	VEN+Ara-C 1个疗程	CR	–	WT1（–）	–	是	存活	10.9

**注** ^a^患者转院进行异基因造血干细胞移植治疗；^b^患者放弃治疗；^c^初次诱导治疗后骨髓形态CR，后失访；^d^采用流式细胞术检测方法，微小残留病（MRD）阴性指MRD水平<10^−4^；VAA+P：VEN+阿扎胞苷/地西他滨+阿柔比星+醋酸泼尼松；V+IA：VEN+伊达比星+阿糖胞苷；Ara-C：阿糖胞苷；CR：完全缓解；OS：总生存

3. 不良反应：VEN联合方案诱导治疗期间最常见的非血液学不良事件是1级或2级恶心呕吐及便秘，所有患者几乎均见。所有患者均有3级或4级血液学不良反应，包括中性粒细胞减少和血小板减少。1例患者在诱导治疗过程中出现难以忍受的疼痛，需要药物干预。4例患者治疗过程中发生凝血功能障碍，纤维蛋白原降低。7例患者出现不同部位的感染，详见表4。所有患者经初始诱导治疗后造血功能恢复且无致死性大出血发生；无患者出现肿瘤溶解综合征及神经系统不良反应；同时无3级及以上脏器不良反应发生（患者治疗前基础病除外）。没有患者因不良反应停药，截至2024年7月21日，13例患者中7例存活，5例死亡，1例失访。其中，4例患者死亡原因均为allo-HSCT后的移植并发症。例7未进行allo-HSCT，因白血病中枢神经系统浸润死亡。

**表4:** 维奈克拉联合多药化疗诱导治疗13例初诊未明系列急性白血病患者的不良反应（例）

不良反应	1级	2级	3级	4级
中性粒细胞减少	0	0	0	13
血小板减少	0	0	0	13
血红蛋白减少	0	0	11	0
发热性中性粒细胞减少	0	0	11	0
肺炎	0	2	0	0
败血症	0	4	0	0
凝血功能障碍	0	4	0	0
恶心	8	4	0	0
呕吐	4	0	0	0
腹泻	3	1	0	0
便秘	1	0	0	0
口腔黏膜炎	1	0	0	0
疼痛	1	0	0	0
心脏疾病	1（阵发性房颤）^a^	0	0	0
脑血管疾病	1（急性脑梗）^b^	0	0	0

**注** ^a^无症状，无需治疗；^b^轻度症状，不需要干预

## 讨论

ALAL是指一部分急性白血病的原始细胞缺乏向单一造血系列分化的特征，发展为一组罕见的、异质性强的恶性血液系统疾病。ALAL患者起病急，现临床病例较少，表现为异常原始细胞浸润导致骨髓正常造血功能受抑制，从而出现贫血、感染、出血等骨髓衰竭表现。ALAL患者在细胞形态学表现上并无明显特异性，甚至有较大一部分病例单从形态学上并不能区分白血病细胞所属系列。其诊断是基于形态学、细胞化学、免疫表型、细胞遗传学和分子生物学结果。

我们对ALAL相关临床和遗传生物学表现的认知较模糊，ALAL也不具备明确的预后因素和治疗分层，在治疗方面缺乏一致性。既往文献报道，ALL样治疗和allo-HSCT改善了ALAL特别是MPAL的结局。一项荟萃分析和系统评价检索了到2017年6月止7个数据库MPAL患者的治疗类型（ALL方案/AML方案/“混合”方案）及疾病反应和患者生存。meta分析显示，ALL样方案治疗与MPAL较高的初始缓解率相关（73.9％对53.5％）[2]。Rasekh等[3]和Lazzarotto等[4]也发现与基于髓系的方案治疗MPAL相比，基于淋系的治疗方案显示出更好的应答率。此外Lazzarotto等[4]提出ALL样方案诱导治疗后MPAL的巩固治疗应尽可能包括allo-HSCT。一些病例也显示ALL样初始诱导化疗或“混合”方案治疗ALAL患者的CR率高于接受基于AML样治疗的患者[5]–[6]，并强调强化方案后的allo-HSCT可能有利于成人ALAL[6]。

VEN在多种血液肿瘤，包括AML、CLL及ALL中已有广泛应用，表现出良好的抗肿瘤作用及安全性，并且已有多篇病例报告表明VEN在ALAL中同样具有疗效[14]–[23]。我们分析既往病例报告中经VEN联合方案治疗的19例ALAL患者的临床资料和治疗效果。19例患者中，B/T系MPAL 2例，T/髓系MPAL 5例，B/髓系MPAL 6例，ALAL-NOS 6例。其中无Ph^+^患者，1例患者存在FLT3-ITD突变[19]。7例患者在首次治疗时使用VEN联合方案，12例在VEN联合方案治疗前存在既往化疗/移植处理。11例患者的VEN联合方案为VEN+去甲基化药物；3例患者的方案为VEN+FLAG（氟达拉滨+阿糖胞苷+非格司亭）；2例方案为VEN+AAVP（阿柔比星+阿糖胞苷+长春新碱+泼尼松）；2例为VEN+HOAP（高三尖杉酯碱+阿糖胞苷+长春新碱+泼尼松）；1例为VEN+克拉屈滨+低剂量阿糖胞苷。19例患者中，2例患者经VEN联合方案治疗后NR[17]，[19]，其余17例患者应用VEN联合方案治疗后均获得复合CR，其中15例达到CR，2例达到CRi[21],[23]。

目前，作为病例数最多的研究，我们纳入并随访了13例初诊ALAL患者，他们均接受了VEN联合多药化疗的诱导治疗及巩固治疗。在诱导治疗的联合方案选择方面，我们针对年轻及一般状况较好的患者（5例）使用V+IA方案，针对年纪较长或体能状态差的患者（8例）使用VAA+P方案治疗，所有13例患者诱导治疗后均达到CR（100％），对于初诊接受VAA+P方案诱导治疗的老年及体能状态差的8例患者，4例老年患者（年龄≥70岁）持续采用原方案巩固治疗（1例巩固治疗前失访），4例患者缓解且体能状态恢复后，尽快行allo-HSCT。以V+IA方案为诱导治疗的5例患者，采用VEN+大剂量阿糖胞苷方案巩固治疗1～2个疗程后，尽快行allo-HSCT。VEN联合多药化疗在ALAL中的治疗优势主要体现在以下几个方面：①所有13例患者诱导治疗1个疗程即获得CR，且获MRD阴性，说明该方案在ALAL患者中具有非常好的敏感性；②13例患者中，除1例失访，3例年龄大于70岁的患者外，其余9例患者均顺利桥接移植，且无复发相关死亡，说明该方案在ALAL中具有很强的缓解深度；③13例患者中，无患者发生早期死亡，治疗相关不良反应可耐受，具有很好的安全性。这提示以VEN为基础的联合化疗方案是ALAL的潜在有效、安全的治疗方案。并且我们认为患者在疾病CR后应尽快进行allo-HSCT。研究表明，相比于相合亲缘供者或相合无关供者HSCT，半相合HSCT因其更高的可获得性和更强的移植物抗白血病效应在MPAL中具有良好的OS和无事件生存[27]。后续我们将进一步设计临床试验验证经VEN联合方案治疗后CR的患者桥接半相合HSCT的有效性及安全性，更好地为治疗这一疾病提供最佳治疗策略。

由于ALAL发病率较低，我们的研究仅纳入了13例患者，且为单中心回顾性分析，因此本研究结果具有部分局限性。后续我们将纳入更多的患者，进行多中心前瞻性队列研究，并延长随访时间，以进一步证实VEN联合多药化疗治疗ALAL患者的疗效和安全性。
